# Evolution of Polycomb-group function in the green lineage

**DOI:** 10.12688/f1000research.16986.1

**Published:** 2019-03-08

**Authors:** Daniel Schubert

**Affiliations:** 1 Department of Biology, Epigenetics of Plants, Freie Universität Berlin, Berlin, Germany

**Keywords:** Polycomb, PRC2, phase transition, telomeres, H3K27me3, plant evolution

## Abstract

Epigenetic gene regulation ensures the mitotically or meiotically stable heritability (or both) of gene expression or protein activity states and maintains repetitive element repression and cellular identities. The repressive Polycomb-group (PcG) proteins consist of several large complexes that control cellular memory by acting on chromatin and are antagonized by the Trithorax-group proteins. Especially, Polycomb repressive complex 2 (PRC2) is highly conserved in plants and animals but its function in unicellular eukaryotes and during land plant evolution is less understood. Additional PcG complexes and associated proteins are only partially conserved and have evolved in a lineage-specific manner. In this review, I will focus on recent advances in the understanding of PcG function in the green lineage and its contribution to land plant evolution.

## Introduction

Polycomb-group (PcG) proteins maintain cellular and tissue identity in multicellular organisms and regulate phase transitions in plants ^1^. As PcG orthologues are absent from *Saccharomyces cerevisiae* and *Schizosaccharomyces pombe* genomes, PcG function has long been thought to be nonessential in unicellular organisms. However, recent genomic analyses have identified orthologues and function of PcG genes in many unicellular organisms which may unravel their ancient function in genome regulation ^2^. As more and more PcG-associated and clade-specific factors have been identified in the green lineage, particularly in embryophyta, PcG function can be studied in relation to land plant evolution and phase transitions occurring in these organisms.

PcG proteins assemble in several large complexes, the Polycomb repressive complexes (PRCs). Particularly, PRC2 subunits are highly conserved, even in some unicellular organisms, and mediate trimethylation of histone H3 lysine 27 (H3K27me3) ^3, 4^. PRC1, a Ring Finger E3 ligase complex which catalyzes the monoubiquitylation of histone H2A, is also likely to be evolutionarily ancient as orthologues of its subunits are found in mammals, plants, insects, and other species but have been lost in more primitive organisms ^3– 5^. PcG proteins repress distinct elements in the genome, ranging from repetitive elements to developmental and stress responsive genes, and have a preference for repetitive elements in more primitive organisms and for genes in more complex organisms ^2, 4– 7^.

Here, I will summarize recent advances in studying PcG function in the green lineage as exciting new analyses uncovered target genes, PcG recruitment factors, and function of PcG proteins in more primitive organisms. These studies shed light on the evolution of PcG function and their contribution to genome and developmental regulation. I do not cover all general aspects of PcG-mediated gene regulation as this has been extensively reviewed recently ^1, 3^.

## Conservation and function of Polycomb-group proteins

Recent analyses have identified PRC2 orthologues and the presence of H3K27me2/3 in various unicellular and primitive organisms such as ciliates ( *Tetrahymena thermophila*), diatoms ( *Phaeodactylum tricornutum*), filamentous fungi ( *Neurospora crassa*), green algae ( *Chlamydomonas reinhardtii*), and red algae ( *Cyanidioschizon merolae*). Obvious orthologues of PRC1 are not present in the genomes of these organisms but are present only in higher animals (Bilateria) and emerged in land plants such as *Marchantia polymorpha* and *Physcomitrella patens* ^2, 5– 9^, suggesting a function for PRC1 in controlling multicellularity or complex tissue organization or both. Generation of genome-wide occupancy maps of the PRC2 mark H3K27me3 or knock-down analyses of PRC2 members (or both) revealed an important function for PRC2 in repressing repetitive elements and heterochromatic regions in the primitive organisms, whereas preferential gene-specific H3K27me3 occupancy is found in the moss *P. patens* and in the flowering plant *Arabidopsis thaliana* ^10, 11^. Nevertheless, in all species examined, the presence of H3K27me3 is correlated with low expression level on a genomic view. In addition, H3K27me3 occupies larger domains of multiple nucleosomes, which is required for its inheritance through cell division. In higher plants ( *Arabidopsis*), the role of H3K27me3 in heterochromatin and repetitive element regulation is likely substituted by additional epigenetic regulators such as ARABIDOPSIS TRITHORAX-RELATED PROTEIN 5 (ATXR5) and ATXR6, which mediate H3K27me1 ^12^.

Importantly, studies in the moss *Physcomitrella* indicate that PRC2 became important to regulate phase transitions as PRC2 mutants display sporophytic traits in the haploid gametophytic phase ^13, 14^. In addition, PRC2 represses the evolutionarily conserved homeobox gene *BELL1*, a master regulator for the gametophyte-to-sporophyte transition in *Physcomitrella* ^15^ and possibly its interaction partner KNOX2, which is an H3K27me3 target ^11, 16^. As a KNOX protein and a BELL-related protein promote zygote formation in the haplontic alga *Chlamydomonas* and PRC2 is present in the *Chlamydomonas* genome ^17^, it will be interesting to investigate whether PRC2 is also controlling phase transitions prior to the emergence of land plants.

Whereas the H3K27me3 “writer” PRC2 is widely conserved, the “reader” of H3K27me3 likely has evolved several times. The first H3K27me3 reader was identified in *Drosophila* as the chromodomain protein POLYCOMB which is probably conserved in most vertebrates and invertebrates ^18, 19^. In *Arabidopsis*, H3K27me3 recruits the related but not orthologous chromodomain protein LIKE HETEROCHROMATIN PROTEIN1 (LHP1) ^20, 21^; however, as *lhp1* mutants have a weak phenotype (in contrast to PRC2 knock-outs), it was hypothesized that additional Arabidopsis proteins have H3K27me3 binding capacity. Indeed, recently, the PHD-domain protein EARLY BOLTING IN SHORT DAYS (EBS) and SHORT LIFE (SHL) were shown to have overlapping function with LHP1 and “read” H3K27me3 ^22– 24^. Interestingly, the most ancient LHP1 homologues are found in land plants, such as *M. polymorpha* and *Physcomitrella*, similar to other PRC1 members ^9, 25^, whereas EBS and SHL are conserved in charophytic algae (such as *Chara braunii*) ^26^ and possibly even in chlorophytic algae (such as *Chlamydomonas* and prasinophytes), suggesting specific inventions or requirements of PRC1 during land plant evolution.

## Recruitment of Polycomb-group proteins

In *Drosophila*, recruitment of PcG proteins is mediated by Polycomb response elements (PREs), DNA elements which maintain gene repression but are diverse in sequence and evolutionarily not conserved ^3, 27^. In *Arabidopsis*, PRE-like elements have recently been identified, suggesting that also in plants a combination of diverse sequences and DNA-binding factors are involved in the recruitment of PcG proteins ^28, 29^. While PRC1 and PRC2 members do not possess DNA-binding motifs, VIVIPAROUS1/ABI3-LIKE1 (VAL1) and VAL2, which are DNA sequence–specific binding factors, directly associate with specific DNA sequence elements of PcG target genes and bind to H3K27me3 and PRC1 members ^30– 32^, suggesting that they are the long-sought mediators between PcG proteins and DNA. Interestingly, similar to other PRC1 proteins, VAL proteins are highly conserved in the land plants *Marchantia* and *Physcomitrella* but are likely absent in charophytic algae ^25, 26^. A novel class of PcG target gene binding factors was recently identified by genetic screening in *Arabidopsis*, the telomere repeat-binding factors (TRBs) which regulate both telomeres and PcG target genes ^29, 33, 34^. Interestingly, H3K27me3 is highly abundant at telomeres, including those of humans, *Arabidopsis*, the red algae *C. merolae*, and the fungus *N. crassa* ^5, 7, 35, 36^, indicating an ancient connection between telomeric sequences/telomeres and PcG recruitment. In *Neurospora*, short arrays of telomeric repeats are sufficient to recruit PRC2/H3K27me3 ^37^. Telomeric repeat-containing RNAs (TERRAs) have been identified in various species, including humans and *Arabidopsis* ^38, 39^, and recently it was revealed that TERRAs bind to PRC2 in humans ^35^. Whether this is a general principle is currently unclear; however, there is increasing evidence that telomeric repeats, either as DNA sequence or as transcript, are involved in recruitment of PcG proteins across kingdoms. It is likely that regulation of telomeres by PcG proteins is evolutionarily more ancient but has been adopted by acquisition of telomeric repeat sequences at genes to recruit PcG proteins.

Although TRB proteins bind to PRC2 members ^27^, they are likely not permanently associated with PRC1 or PRC2 as TRB1 complex isolation analyses did not identify these proteins ^40^. However, TRB1 likely forms a complex with PcG-associated proteins, ENHANCER OF POLYCOMB RELATED1/2 (EPCR1/2) and PWWP-DOMAIN INTERACTOR OF POLYCOMBS1 (PWO1) ^40, 41^. While PWO1 shows protein–protein and genetic interactions with PRC2 genes ^41^, the connection of EPCR1 with PcG is less clear, especially as these proteins, together with PWO1 and TRBs, also regulate heterochromatin silencing and telomere length ^40^. Thus, these proteins and complexes likely have a more general role in chromatin silencing but may be important for stabilizing PcG silencing or creating a repressive environment.

## Conclusions

Recent analyses, particularly in *Arabidopsis*, have resolved long-standing questions in PcG regulation in plants, including the isolation of novel H3K27me3 readers and the identification of PRC1-like complexes and PRE-like recruitment elements. It is apparent that PRC2 has fundamental functions as it is highly conserved, even in unicellular eukaryotes. Although it targets and regulates repetitive elements and telomeric sequences in these species, it also targets genes which are largely not repressed but may be inducible upon environmental change or activated in a different phase of the life cycle. As PcG proteins regulate phase transitions in seed plants and mosses by controlling specific transcription factors and all of these factors are conserved in unicellular and multicellular algae, it is tempting to speculate that the transition from the haploid to the diploid phase (and vice versa) in these organisms is regulated by PcG. Thus, genetic analyses in these algae and primitive land plants such as *Marchantia* will be required to elucidate these questions.

While expression of PcG genes is largely not transcriptionally regulated, it will be interesting to reveal whether level or activity of these proteins is regulated post-transcriptionally in a developmental and responsive manner. Interestingly, a recent analysis of cold responses and circadian rhythms in *Arabidopsis* uncovered extensive alternative splicing of several PcG mRNAs likely leading to an altered PcG proteome ^42^.

Overall, it will be highly interesting and rewarding to study PcG function in non-seed plants to learn about land plant evolution, regulation of phase transitions, the evolution of multicellularity and distinct cell types, and genome regulation in general. While plants have evolved novel innovations in relation to PcG function, the general principles and factors involved appear to be higher conserved than previously anticipated but are employed for different gene regulatory functions.

## Abbreviations

ATXR, ARABIDOPSIS TRITHORAX-RELATED PROTEIN; EBS, EARLY BOLTING IN SHORT DAYS; EPCR1/2, ENHANCER OF POLYCOMB RELATED1/2; H3K27me3, histone H3 lysine 27 trimethylation; PcG, Polycomb group; PRC, Polycomb repressive complex; PRE, Polycomb response element; PWO1, PWWP-DOMAIN INTERACTOR OF POLYCOMBS1; SHL, SHORT LIFE; TERRA, telomeric repeat-containing RNA; TRB, telomere repeat-binding factor; VAL, VIVIPAROUS1/ABI3-LIKE
